# Caesarean delivery in the Limbé and the Buea regional hospitals, Cameroon: frequency, indications and outcomes

**DOI:** 10.11604/pamj.2016.24.227.9499

**Published:** 2016-07-13

**Authors:** Tanyi John Tanyi, Julius Atashili, Peter Nde Fon, Tchounzou Robert, Koki Ndombo Paul

**Affiliations:** 1 Department of Paediatrics and Obstetrics/Gynecology Limbé Regional Hospital, Limbe, Cameroon; 2 Department of Public Health and Hygiene, Faculty of Health Sciences, University of Buea, Buea, Cameroon; 3 Department of Obstertrics/Gynecology, Limbé Regional Hospital, Limbe, Cameroon; 4 Department of paediatrics, Faculty of Medicine and Biomedical Sciences, University of Yaounde, Yaounde, Cameroon

**Keywords:** Caesarean delivery, adverse neonatal outcome, prevalence, resource-limited settings

## Abstract

**Introduction:**

Neonatal outcomes can be directly and indirectly affected by caesarean delivery (CD). Data on CD rates in semi-urban and rural hospitals in resource-limited settings are scarce and yet are needed to better guide the care of women and neonates in these settings. we carried out this study to determine the frequency of CD, its indications and the frequency of the various adverse neonatal outcomes (ANO) in the Limbe Regional Hospital (LRH) and the Buea Regional Hospital (BRH), Cameroon. We also assessed the relationship between the indication for CD and ANO in the said hospitals.

**Methods:**

This was a hospital-based retrospective and prospective cross-sectional study using descriptive and analytic methods conducted in the LRH and the BRH maternity units within a nine months period in 2015. Informed consent was obtainedfrom mothers of the neonates. Data analyses were performed using Epi-Info 3.5.4 software.

**Results:**

We recruited 199 neonates born through CD. The prevalence of CD was 13.3% with cephalopelvic disproportion (CPD) being the most frequent (32.2%) indication for CD. There were 52 (26.1%) ANO following CD and respiratory distress was the most common 24 (46.2%) of all ANO. Emergency indications for CD were associated with more ANO 49 (34.5%) as compared to elective indications for CD 3 (5.3%) [p-value<0.001]. We noted a significant association between indications for CD and the various type of ANO, with CPD having the worse prognostic neonatal outcomes 30.8% [p-value=0.02].

**Conclusion:**

The prevalence of ANO associated with CD in our hospitals was high with a worrying prognosis. While the exact reasons are unknown, the creation of well-equipped neonatal units with trained staff, may contribute to reduce neonatal morbidity and fatalities. Furthermore, the association of CPD to worse prognostic neonatal outcomes calls for clinicians, to consider additional management options, such as antibiotic prophylaxis and oxygen therapy to the neonates, prior to CD.

## Introduction

Worldwide rise in caesarean delivery (CD) rates during the last three decades has been a cause of alarm [1]. The rates of such delivery have increased dramatically in recent years, from 12% in 1990 to 24% in 2008 with no improvement in outcome for neonates [2]. The increasing rates of CD have been debated globally for over two decades, especially during the 1980s, when a peak was reached in industrialized Countries [3]. This lead to guidelines from the relevant United Nations (UN) agencies for CD rates in a country, it stated that; CD rates should be between 5% and 15% [4, 5] and further research on the issue was warranted.

In 2002, more than one-fourth of all births (26.1%) in United States were by CD, the highest ever reported rate [6]. In 2004, rate of births from CD, for first pregnancies increased to 29.1% of all births, continuing a rising trend [6], with labour dystocia, previous CD, breech presentation and fetal distress being the most common indications of CD amongst others. Babies born via caesarean delivery face more risks than babies born vaginally: they are more likely to have respiratory problems in the neonatal period, more likely to have difficulties establishing breastfeeding, and more likely to experience asthma in childhood and adulthood [7]. Babies delivered after a general anaesthesia have lower Apgar scores than those delivered after spinal anaesthesia [2]. Torkanetal [8], showed that, there is a higher incidence of low Apgar scores, need for resuscitations, birth injury, pulmonary hypertension and respiratory distress amongst neonates born through CD. Worldwide trends towards increased CD rates have also been observed in Africa. The rate of CD in a teaching hospital in Nigeria rose from 7.2% in 2000 to 11.8% in 2009 [9]. Furthermore, the course of increased CD rates has also been noted in Cameroon. The rate of CD in Cameroon varies between 2% and 3% of all deliveries, with higher rates reported for the main teaching hospital (CHU) in Yaoundé, and for the Central Hospital Yaoundé [10]. Also, in a study carried out by Forsah [11], in Buea, Cameroon the CD rate was 23.8%. Amongst the indications of CD; fetal distress, labour dystocia, breech presentation, multiple gestation and previous CD being the most common indications.

A study by Tebeuet al [12], found the poor foetal outcome of foetus delivered through CD in the Far North Region of Cameroon and revealed that one of three caesarean deliveries ended in foetal death. Furthermore Forsah [11], in Buea had a 14.4% Adverse Neonatal Outcome (ANO) following indication for CD. These ANO included respiratory distress, neonatal infections and neonatal death. Data on CD rates and their outcomes guide obstetricians and pediatricians in the management of pregnancy and it outcomes. Unfortunately, such data are rare for semi-urban areas in developing countries. We thus carried out this study to determine the frequency of CD, its indication and the frequency of the various adverse neonatal outcomes (ANO) in the Limbe and Buea Regional Hospitals, two second-level referral hospitals in semi-urban Cameroon. We also assessed the relationship between the indication for CD and ANO in the said hospitals.

## Methods

### Study design and setting

A hospital-based retrospective and prospective study using descriptive and analytic methods was carried out in the Limbé and Buea regional hospitals. These health facilities are located in the South West Region of Cameroon and serves as a second-level referral level maternities in the South West Region. About 160 deliveries are conducted each month amongst which are caesarean deliveries. The maternity units have a total of 50 beds. Both hospitals have qualified and experienced staffs: 5 Doctors (obstetrician/gynecologist, pediatrician), 4 Midwife, 10 Nurses and Birth attendants

### Study population, sampling and study procedures

The study population was made of pregnant women who had delivered by CD and their neonates. A purposive sampling method was used. Any pregnant woman delivered by CD in the LRH and the BRH admitted in the hospitals maternity during the study period were eligible to take part for the prospective phase, while for the retrospective phase, delivery files were reviewed and only files with completed data (that is files with basic demographic and identification data, gestational age, type and indication for CD, and basic clinical examination of neonate) were used. The retrospective phase covered files from July 2015 to December 2015. For the prospective phase we used purposive sampling technique to approach and seek consent from eligible participants admitted in the hospital maternity using a consent form. Questionnaires were presented to those who consented to participate. Record(s) of participants involved in the study were reviewed and the following outcomes amongst neonates born by CD were recorded: the presence or not of respiratory distress, diagnosed based on Silverman-Anderson retraction score (i.e. a score >6) or Downes' Score (i.e. a score>6) [13]; neonatal infection diagnosed based on fever (temperature instability), poor feeding, seizures (seizure was graded using the Blantyre coma scale (i.e. a grade ≤4) [14]); birth injuries diagnosed based on joint dislocation, bone fracture, laceration [15]; the degree of resuscitation based on APGAR score at 1and 5 minute (i.e. a score<6) resuscitation with prolong ventilation (i.e> 5 minutes) [16]; and neonatal death based on brain death guides by Wijdicks [17].

### Data management and data analysis

Data were collected and detailed recording of the types of CD, indications for CD and neonatal outcomes was done. To minimize errors from handling and filling the data sheet, one copy of the data sheet for each patient was filled on hard copy and another was keyed into the EPI info 3.5.4 database in the investigator's computer. 10% of entered questionnaires were double checked. Data were analyzed using Epi-Info 3.5.4 software. Numeric results were presented to the nearest two decimal places. Descriptive statistics (age, gestational age, place of delivery, marital status, type of delivery, type of anaesthesia, gender of neonates, neonatal outcomes, APGAR score, ANO and birth weight) were presented using absolute numbers, means and percentages. Proportions of outcome variables within categories of predictor variables were computed and compared using the Chi- square or Fisher's exact statistical tests where appropriate. Two-tailed p-values ≤ 0.05 were considered statistically significant.

### Ethical Considerations

Administrative approvals were obtained from the South West Regional Delegation of Public Health, the BRH and the LRH. Ethical approval was obtained from the Institutional Review Board of the Faculty of Health Sciences, University of Buea, Cameroon. Written informed consent was obtained from all mothers (caretaker) of the neonates who were enrolled in the prospective phase of the study while a waiver of consent was granted for the retrospective phase of the study by the Institutional Review Board, as it would have been impossible to conduct the study if consent was to be sought from participants but the patient records/information was anonymized and de-identified prior to analysis. To reduce the inconvenience to neonates, clinical examination of neonates were done using standard sterile and temperature-controlled conditions

## Results

**Figure 1:**
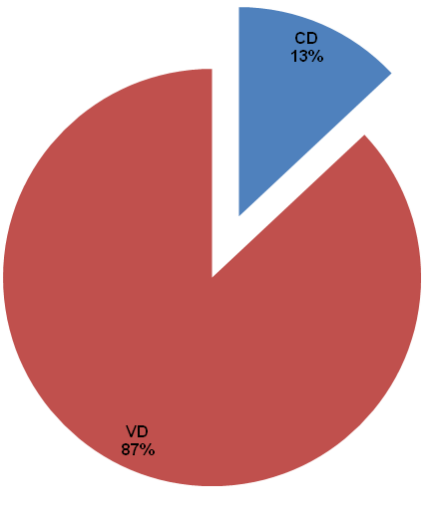
Route of delivery of 1492 neonates in the Limbe and Buea regional hospitals, Cameroon. (CD: Caesarean delivery; VD: vaginal delivery)

**Figure 2:**
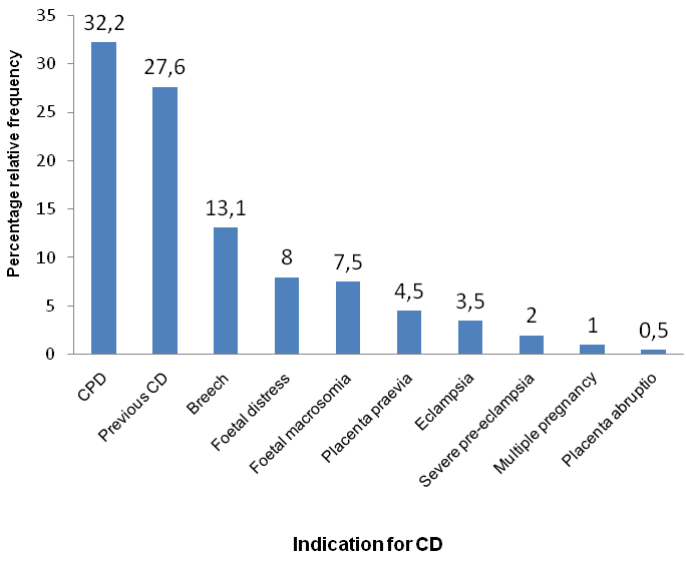
Frequency of indications for caesarean delivery amongst 199 studied participant in the Limbé and Buea regional hospitals, Cameroon

**Figure 3:**
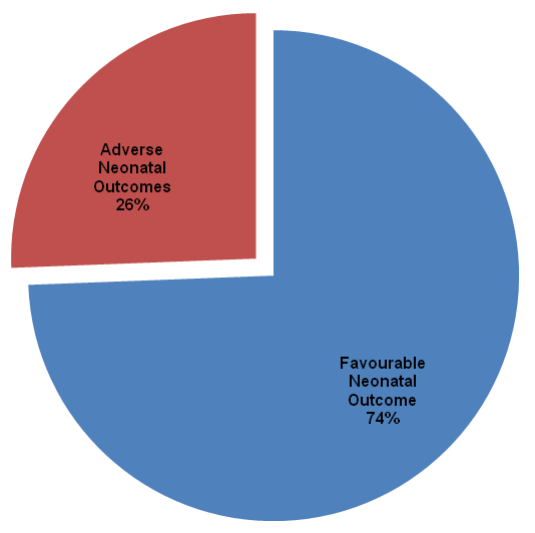
Overall neonatal outcomes following caesarean delivery in the Limbé and Buea regional hospitals, Cameroon

Of the 1492 deliveries conducted during the study period, 199 were caesarean, giving an overall CD rate of 13.3% (Figure 1). The characteristics of the 199 women and their neonates included in this study and the outcomes of CDs are summarized in Table 1, Table 2. Most of our participants (58%) were in the age range between 25-34 years, majority (70%) had gestational age ranges between 38-41 weeks, 67% delivered in the BRH. As many as 83% of CDs were done under General anesthesia, and 71% were emergency CDs. Cephalo-pelvic disproportion (CPD) was the most frequent (32.2%) indication for CD (Figure 2). Also, of the 199 neonates born, 52 (26.1%) had at least one adverse neonatal outcomes (ANO), with respiratory distress being the most frequent (46.2%) ANO (Figure 3). Table 3 shows the association between the indications for CD and the various types of ANO. There was a statistical significant relationship between ANO and indication for CD, p-value=0.02 using Fisher's exact test (Table 3). Also, ANO were significantly more frequent following emergency CDs compared to elective CDs, p-value=<0.001 using Chi-square test (Table 4).

**Table 1:** Characteristics of 199 women and neonates included in a study of Caesarean delivery in the Limbe and Buea regional hospital, Cameroon

Characteristic	Level	n	%
**Age (years)**	15-24	67	34
	25-34	116	58
	35-44	8	16
**Marital status**	Married	144	72
	Single	55	28
**Gestatiionalage (weeks)**	30-33	8	4
	34-37	32	16
	38-41	139	70
	42	20	10
**Place of delivery**	LRH	65	33
	BRH	134	67
**Gender of neonate**	Male	114	57
	Female	85	43
**Type of anaesthesia**	General anaesthesia	166	83
	Spinal anaesthesia	33	17

**Table 2:** Characteristics of 199 caesarean deliveries and their outcomes in the Limbe and Buea regional hospital, Cameroon

Characteristic	Level	n	%
Type of CD	Emergency	142	71
	Elective	57	29
Outcome of CD	Favourable	147	74
	ANO	52	26
Types of ANO	Respiratorydistress	24	46.2
	Neonataldeath	15	28.8
	Infection	8	15.4
	Resuscitation	4	7.7
	Birthinjury	1	1.9

**Table 3:** Association between indications for Caesarean Delivery and the various types of ANO

	TYPES OF ADVERSE NEONATAL OUTCOME						
Indication for CD	RD n(%)	n(%) ND	Infection n(%)	RE n(%)	BI n(%)	TOTAL n(%)	P value
CPD	6(25.0)	6(40.0)	2(25.0)	2(50.0)	0(0.0)	16(30.8)	
Foetal Distress	5(20.8)	2(13.3)	1(12.5)	2(50.0)	0 (0.0)	10(19.2)	
Previous CD	5(20.8)	1(6.7)	2(25.0)	0(0.0)	0(0.0)	8(15.4)	
Breech	0(0.0)	2(13.3)	2(25.0)	0(0.0)	0(0.0)	4(7.7)	
FoetalMacrosomia	2(8.3)	1(6.7)	0(0.0)	0(0.0)	1(100)	4(7.7)	
Placenta Praevia	2(8.3)	2(13.3)	0(0.0)	0(0.0)	0(0.0)	4(7.7)	
Eclampsia	2(8.3)	0(0.0)	1(12.5)	0(0.0)	0(0.0)	3(5.8)	
Severe Pre-eclampsia	1(4.2)	1(6.7)	0(0.0)	0(0.0)	0(0.0)	2(3.8)	
Multiple Pregnancy	1(4.2)	0(0.0)	0(0.0)	0(0.0)	0(0.0)	1(1.9)	
Placenta Abruptio	0(0.0)	0(0.0)	0(0.0)	0(0.0)	0(0.0)	0(0.0)	
TOTAL	24(100)	15(100)	8(100)	4(100)	1(100)	52(100)	0.02

RD=Respiratory Distress, ND=Neonatal Death, BI=Birth Injury, RE= Some Degree of Resuscitation

**Table 4:** Association between adverse neonatal outcomes and types of CD

	Adverse Neonatal Outcome			
Type of CD	YES (%)	NO (%)	TOTAL	P-VALUE
Emergency	49 (34.5)	94 (65.5)	142	
Elective	3 (5.3)	54 (94.7)	57	
Total	52 (26.1)	147 (73.9)	199	<0.001

## Discussion

In this study, we had a prevalence for caesarean delivery (CD) within expected ranges. Cephalopelvic disproportion (CPD) was the most common indication for CD. Furthermore we noted a high prevalence of adverse neonatal outcomes(ANO)with respiratory distress being the most frequent adverse neonatal outcome. There was also a significant association between indications for CD and the various types of ANO. The prevalence of CD was 13.3%. This prevalence falls in the range of 10 -15% as proposed by World Health Organisation (WHO) for the worldwide CD rate [18] and within ranges as reported in other studies [9, 19], the rate is however, lower than the rate observed in the study conducted by Forsah in Buea [11] and in other studies [20–33]. Doctors within our hospitals may be conscious of the WHO proposal that there is no justification for any region to have a CD rates higher than 10-15%, [18, 34, 35]. Also, much awareness is being made to ensure that the ranges of worldwide rate of CD as proposed by WHO [18], is met in SWR. Variation in studies could be attributed to multiple factors including: Maternal demands for the procedure, pressure of obstetrician favouring recourse to CD, changes in size of the population, socioeconomic factors and the limited use of vaginal instrumental delivery.

CPD was the most frequent indication for CD (32.2%). This findings correlates with that made by Forsah in Buea, Cameroon [11], Nana et al [36] in the Far-North region of Cameroon and Sugewe [31] in Yaoundé, Cameroon. It is also consistent with findings in many other studies out of Cameroon [9, 20, 23, 24, 33, 37–40]. The high frequency of CPD as indication was in contrast with reports of a previous CD being the most common indication in studies by Aziz et al in Pakistan [21] and Bangalet al in India [22]. In our study previous CD was only the third indication suggesting that obstetricians in our setting may not necessarily be adhering to the dictum that 'once a CD always a CD' [41, 42].

In this study, nearly one in every five neonates born through CD had an Adverse Neonatal Outcome (ANO). This correlates with findings made by Forsah in Buea, Cameroon [11] and Tebeuet alin Northern Cameroon [12]. This could be because most of the caesarean deliveries were done as emergencies, the relatively frequent use of general anesthesia and the lack of Neonatal Intensive Care Unit (NICU). Other studies have reported that increased use of general anesthesia in CDs was associated with increased ANO [2, 43–45]. Furthermore, in a study carried out by Nana et al in the Far-North Region of Cameroon, revealed that emergency indication for CD was associated with high ANO [36]. The absence of paediatricians and neonatologists may contribute to these high rates of ANO Respiratory Distress was the most common ANO identified (>30%). This is consistent with findings in other studies [19, 38, 46, 47]. On the other hand, in studies conducted by Allison in Australia [48] and Tebeuet al in Northern Cameroon [12], neonatal death (which is the second most frequent ANO in our study) was the most common ANO. This variation could be related to the different indications for CD: In the study by Allison in Australia [48] breech presentation was the most frequent indication for CD while “extreme ages of reproductive life” was the most frequent indication noted by Tebeuet al in Northern Cameroon [12].

In this study, there was an association between the types of CD and ANO. We observed more ANO (34.5%) with Emergency indications for CD. Similar findings were observed by Forsah and Tebeuet al in Cameroon [11, 12] and Zanardoet al in Italy [49]. The high rates of ANO could be due to the exigencies of the emergency indication for CD. There was also a significant association between the indications for CD and the various types of ANO with CPD emerging with the most (30.8%) types of ANO (infection, neonatal death, respiratory distress, degree of resuscitation), closely followed by foetal distress (19.2%), with similar ANO. It is also, worth noting that, placenta abruptio, had no risk for ANO, closely followed by; multiple pregnancy with 1(1.9%) ANO (Respiratory distress). This could be related to the high prevalence of CPD (32.2%) and low prevalence of multiple pregnancy (1%) and placenta abruptio (0.5%) observed in our study.

This study was limited to just two hospitals within Cameroon. Nevertheless result from our study are similar to those of other previous studies [9, 18, 19, 23, 33, 38, 39, 49]. Also, we limited this study to the short term outcomes following CDs. Future studies will need to assess the long term outcomes of CD in our milieu. Furthermore, other studies will need to compare the frequencies of adverse outcomes between neonates born via caesarean and neonates born via the vaginal route.

## Conclusion

This study has shown that, the prevalence of CD (13.3%) is within the range acceptable toWHO, with CPD being the most frequent (32.2%) indication for CD. Moreover, at least one in every five neonates born through CD had an ANO with respiratory distress being the most frequent ANO. Furthermore, there was a significant association between indications for CD and the various types of ANO. CD for CPD had the worse prognosis of ANO. The CD rate combined with the high rates of adverse outcomes is a call for concern. Health personnel will need to be made aware and trained on the management of neonates born of CDs in this setting. The training ought to also reinforce the need for close monitoring of labour, through the appropriate use of a partogramme. This also allow for early detection of the need for a CD Furthermore, the technical capabilities of health facilities will need to be re-enforced to foster neonatal care.

### What is known about this topic

- The rate of CD;
- Indications of CD.

### What this study adds

- At least one in every five neonates born through CD had an ANO;
- A significant association between indications for CD and the various types of ANO;
- CD for CPD had the worse prognosis of ANO.
